# Social Determinants of Successful Smoking Cessation: An Eight-Year Analysis of Population Assessment of Tobacco and Health (PATH) Data

**DOI:** 10.31586/jbls.2024.1070

**Published:** 2024-10-30

**Authors:** Shervin Assari, Payam Sheikhattari

**Affiliations:** 1 Charles R Drew University of Medicine and Science, Los Angeles, CA, USA; 2 Morgan State University, Baltimore, MD, USA

**Keywords:** Smoking Cessation, Health Disparities, Racial/Ethnic Differences, Education, Social Determinants, Ethnic Groups

## Abstract

**Background:**

Smoking cessation is a crucial public health goal due to its substantial impact on reducing the morbidity and mortality associated with tobacco use. However, significant disparities in smoking cessation success persist across socioeconomic groups in the United States.

**Objectives:**

This study aimed to examine differences in smoking cessation rates among daily smokers based on race, ethnicity, and socioeconomic status (SES) using data from the Population Assessment of Tobacco and Health (PATH) study, spanning waves 1 to 6 (eight years).

**Methods:**

Longitudinal data from PATH were analyzed, focusing on baseline daily cigarette smokers followed over an eight-year period to assess cessation outcomes. SES was measured by education and poverty status. Successful smoking cessation was defined as sustained abstinence from cigarettes for 12 months or more at the final wave. Logistic regression models identified predictors of successful cessation, adjusting for potential confounders, including age, nicotine dependence, and access to cessation resources.

**Results:**

The analysis revealed significant disparities in cessation success across racial, ethnic, and SES groups. Smokers living in poverty and those with lower educational attainment were less likely to achieve cessation success than their counterparts. Race (Black) and ethnicity (Latino) were also significantly associated with lower cessation success.

**Conclusions:**

This study highlights the social determinants of smoking cessation success among U.S. adult smokers, with lower success rates observed among those in poverty and with less educational attainment. These findings emphasize the need for targeted interventions that address the unique barriers to cessation faced by low-SES groups. Public health strategies should prioritize equitable access to cessation resources and culturally tailored interventions to reduce these disparities and improve cessation outcomes among all smokers.

## 1. Introduction

Smoking cessation is a critical public health goal due to its significant impact on reducing morbidity and mortality associated with tobacco use [1]. Despite progress in reducing smoking rates in the U.S. over the past several years, cigarette smoking remains one of the leading causes of death, largely due to its role in a wide range of chronic diseases, including cardiovascular disease, cancer, and respiratory illnesses [2–4]. Although various cessation aids—such as nicotine replacement therapies, prescription medications, and behavioral interventions [5–7]—have proven effective, overall quit success rates remain suboptimal. The benefits of successful cessation extend beyond individual health improvements; they also reduce smoking-related healthcare costs, benefiting society as a whole [8–10]. However, the success of quitting is not evenly distributed across the population [11,12], with significant disparities in smoking cessation rates among different sociodemographic groups [13–15]. Marginalized populations defined by race, ethnicity, and socioeconomic status are at higher risk of unsuccessful tobacco quit attempts [16,17]. Understanding these disparities is crucial for guiding investments toward developing targeted interventions that can effectively support racialized and minoritized smokers in their cessation efforts [18–22]

A pressing challenge in smoking cessation research is the persistent disparities in quit rates among Black, Latino, and low socioeconomic status (SES) individuals [23]. These disparities have remained pronounced over the years in the U.S. For example, evidence from the PATH study and the Centers for Disease Control and Prevention (CDC) [24,25] indicates that Black, Latino, and lower-educated smokers are less likely to successfully quit smoking compared to their White, non-Latino, and highly educated counterparts [26,27]. These disparities are not simply due to individual differences in motivation, character, or willpower; rather, they are deeply rooted in broader social, economic, and structural factors that shape access and conditions for quitting. Racial and ethnic minorities, as well as individuals with lower educational attainment, often face unique stressors, such as discrimination, reduced access to healthcare resources, and residence in areas with limited cessation services, all of which can hinder their ability to quit smoking successfully. Additionally, these groups are more likely to experience higher levels of nicotine dependence, receive less support for quitting, and be exposed to environments that promote smoking, all of which increase the difficulty of achieving successful cessation [28,29]. Addressing these disparities is not only a matter of equity but also a crucial component of achieving broader public health goals in the U.S., such as reducing overall smoking rates and associated healthcare costs.

### 1.1. Aims

The primary objective of this study is to explore the factors associated with successful smoking cessation among daily cigarette smokers over an eight-year period using data from the Population Assessment of Tobacco and Health (PATH) [30–32] study. Specifically, the study aims to identify demographic groups that are less likely to quit smoking successfully, focusing on Black individuals, Latino individuals, those living in poverty, and those with lower educational attainment. This focus is driven by the recognition that these groups have historically faced significant barriers to smoking cessation, including but not limited to limited access to healthcare resources, higher levels of nicotine dependence, and exposure to targeted tobacco marketing. By understanding these barriers, the study seeks to inform the development of more effective, targeted interventions to support smoking cessation efforts among these marginalized populations.

## 2. Methods

### 2.1. Study Design

This study utilizes data from the Population Assessment of Tobacco and Health (PATH) study [30–32], a nationally representative longitudinal cohort study of U.S. adults that assesses tobacco use and its health effects. The analysis was limited to participants who were current cigarette users at baseline, defined as daily users or those who smoked on some days per week. The study followed these participants over eight years, across waves 1 to 6, to assess smoking cessation outcomes.

### 2.2. Sample and sampling

The PATH study employs a complex survey design, including stratification, clustering, and weighting, to ensure that the results are representative of the U.S. adult population.

### 2.3. Outcome Measure

The primary outcome of interest was successful smoking cessation, defined as self-reported sustained abstinence from cigarette smoking as assessed at the final wave (wave 6; final data collection). Participants were considered successful quitters if they were current smokers at baseline (wave 1; initial data collection) but reported being former smokers at wave 6, indicating they had maintained abstinence from smoking for the required period to be classified as having successfully quit.

### 2.4. Predictors and Covariates

The main predictors examined in this study were race (Black, White, Other), ethnicity (Latino, non-Latino), and health insurance status (insured, uninsured, type of insurance). Socioeconomic status was measured using these measures: education level (categorized as less than high school, GED, High school graduate, some college (no degree) or associate, Bachelor’s degree, and advanced degree) and poverty status (below or above the federal poverty line). Covariates included age (a 7-level categorical variable), sex (male or female), and region of the country (Northeast, Midwest, South, and West) to adjust for demographic differences and potential confounding factors.

### 2.5. Statistical Analysis

All statistical analyses were performed using Stata version 18.0, with the survey design features activated to ensure accurate variance estimation and representative results for the U.S. adult smoking population. We employed the survey (svy) commands to account for the complex sampling design of the PATH study, including stratification, clustering, and weighting. As such, weighted proportions instead of non-weighted n were reported. Logistic regression models were used to assess the association between the predictors (race, ethnicity, education, poverty status, and insurance status) and the likelihood of successful smoking cessation over the eight-year follow-up period. The survey weights were applied to produce estimates that are representative of the U.S. population of smokers. The primary model included all main predictors and covariates (age, sex, and region). Odds ratios (ORs) and 95% confidence intervals (CIs) were calculated to estimate the strength and precision of the associations between the predictors and the outcome. Interaction terms between race, ethnicity, and education were tested to explore potential differential effects across these groups. Model diagnostics, including checks for multicollinearity and influential observations, were performed to ensure the robustness of the findings.

### 2.6. Ethical Considerations

The PATH study data are publicly available through the ICPSR data and were collected in accordance with ethical standards and regulations, including obtaining informed consent from all participants. This analysis was conducted using de-identified data, and therefore, additional ethical review was not required.

## 3. Results

### 3.1. Descriptive Data

Table 1 presents the descriptive characteristics of current smokers at baseline who were followed for eight years in the PATH study. The sample was geographically distributed across the four Census regions, with the highest proportion of smokers residing in the South (40.50%, 95% CI: 37.91–43.14), followed by the Midwest (24.00%, 95% CI: 21.60–26.58), the West (18.96%, 95% CI: 16.99–21.11), and the Northeast (16.54%, 95% CI: 14.34–19.00).

Age distribution among participants showed that the majority were between 25 to 34 years old (24.08%, 95% CI: 23.19–25.01), followed by those aged 45 to 54 years (19.46%, 95% CI: 18.68–20.27), and 35 to 44 years (18.95%, 95% CI: 18.11–19.82). The youngest group (18 to 24 years old) constituted 15.48% (95% CI: 14.73–16.27), while older age groups were less represented, with 15.06% (95% CI: 14.27–15.89) aged 55 to 64 years, 5.51% (95% CI: 5.03–6.03) aged 65 to 74 years, and only 1.45% (95% CI: 1.21–1.75) being 75 years or older.

In terms of sex, the sample was predominantly male (55.79%, 95% CI: 54.87–56.71), with females comprising 44.21% (95% CI: 43.29–45.13). Racial distribution showed that a large majority of participants were White (75.11%, 95% CI: 72.20–77.81), followed by Black individuals (16.34%, 95% CI: 13.95–19.05), and those classified as Other races (8.55%, 95% CI: 7.57–9.64). Latino ethnicity was reported by 13.29% (95% CI: 11.66–15.11) of participants.

Educational attainment varied, with the largest group having some college education or an associate degree (32.77%, 95% CI: 31.71–33.85). High school graduates made up 28.55% (95% CI: 27.45–29.68), while those with less than a high school education accounted for 16.82% (95% CI: 15.95–17.73). Participants with a GED represented 10.68% (95% CI: 10.01–11.39), those with a bachelor’s degree made up 8.57% (95% CI: 7.80–9.41), and a small proportion had advanced degrees (2.61%, 95% CI: 2.27–2.98).

Regarding poverty status, 39.59% (95% CI: 38.14–41.07) of participants were living below the federal poverty line, while 60.41% (95% CI: 58.93–61.86) were not. Health insurance coverage varied, with 49.06% (95% CI: 47.72–50.40) having some form of private insurance, 8.92% (95% CI: 8.25–9.64) having private insurance with some Medicare, and 13.00% (95% CI: 12.10–13.95) having private insurance or Medicare with some other forms of coverage. Those with only other forms of insurance comprised 3.44% (95% CI: 3.09–3.84), and 25.58% (95% CI: 24.58–26.61) reported having no insurance at all.

Over the eight-year follow-up period, only 8.83% (95% CI: 8.23–9.47) of participants successfully quit smoking, defined as sustained abstinence from cigarette use, while a substantial 91.17% (95% CI: 90.53–91.77) did not achieve successful cessation.

## 4. Logistic Regression

Table 2 presents the results of the logistic regression analysis examining predictors of successful smoking cessation among current smokers at baseline over an eight-year follow-up period. The analysis included various demographic and socioeconomic predictors, with the outcome of interest being sustained abstinence from cigarette smoking at the final wave. Race and ethnicity showed varied effects. Black smokers had significantly higher odds of quitting compared to White smokers (OR = 1.23, 95% CI: 1.03–1.47, p = 0.020), while there was no significant difference for those categorized as Other races (OR = 1.00, 95% CI: 0.76–1.31, p = 0.987). Latino smokers were significantly more likely to quit than non-Latino smokers (OR = 1.66, 95% CI: 1.35–2.03, p < 0.001). Educational attainment was positively associated with cessation success. Compared to those with less than a high school education (reference group), those with a bachelor’s degree (OR = 1.82, 95% CI: 1.35–2.44, p < 0.001) or an advanced degree (OR = 1.71, 95% CI: 1.10–2.66, p = 0.018) had significantly higher odds of quitting. Smokers with some college education or an associate degree had marginally higher odds (OR = 1.22, 95% CI: 0.98–1.51, p = 0.081), though not statistically significant, while those with a GED (OR = 0.94, 95% CI: 0.72–1.24, p = 0.669) and high school graduates (OR = 1.12, 95% CI: 0.89–1.39, p = 0.330) did not show significant differences. Poverty was associated with a lower likelihood of cessation, with individuals below the federal poverty line having lower odds of quitting (OR = 0.84, 95% CI: 0.73–0.97, p = 0.020). Health insurance status did not significantly predict cessation success. Compared to those with some private insurance (reference group), the odds ratios were: private insurance with some Medicare (OR = 0.87, 95% CI: 0.65–1.18, p = 0.375), private insurance or Medicare with other forms (OR = 0.84, 95% CI: 0.67–1.04, p = 0.109), other insurance only (OR = 1.01, 95% CI: 0.68–1.51, p = 0.966), and no insurance (OR = 0.88, 95% CI: 0.75–1.04, p = 0.124). Regarding geographic region, compared to smokers in the Northeast (reference group), those in the Midwest (OR = 1.15, 95% CI: 0.91–1.44, p = 0.238) and South (OR = 1.14, 95% CI: 0.90–1.43, p = 0.272) did not show significant differences in cessation success. Smokers in the West had a higher, though not statistically significant, likelihood of quitting (OR = 1.27, 95% CI: 0.98–1.63, p = 0.067). Age was a significant predictor of cessation success, with younger age groups more likely to quit. Compared to smokers aged 18 to 24 years, older age groups had progressively lower odds of successful cessation. The odds ratios for age groups were: 25 to 34 years (OR = 0.72, 95% CI: 0.61–0.86, p < 0.001), 35 to 44 years (OR = 0.55, 95% CI: 0.45–0.68, p < 0.001), 45 to 54 years (OR = 0.43, 95% CI: 0.34–0.54, p < 0.001), 55 to 64 years (OR = 0.46, 95% CI: 0.35–0.59, p < 0.001), 65 to 74 years (OR = 0.40, 95% CI: 0.26–0.62, p < 0.001), and 75 years or older (OR = 0.22, 95% CI: 0.05–0.97, p = 0.046). Sex also played a significant role, with males having lower odds of quitting compared to females (OR = 0.81, 95% CI: 0.68–0.96, p = 0.018).

## 5. Discussion

Smoking cessation among diverse socioeconomic and demographic groups is a complex phenomenon [13,27,29,33], with differences in successful cessation rates often observed based on race, ethnicity, poverty status, and educational attainment. Various groups may encounter unique barriers that contribute to lower cessation success, such as limited access to cessation aids, cultural factors, and systemic inequities. Based on existing literature, we hypothesize that Black individuals, Latino individuals, those living under poverty, and those with lower educational attainment will exhibit lower rates of successful smoking cessation over the eight-year follow-up period. These hypotheses are rooted in the recognition of both individual-level and structural factors that disproportionately affect these groups, including higher nicotine dependence, less access to support, and exposure to pro-smoking environments.

Community-Based Participatory Research (CBPR) and community-engaged solutions are essential for addressing the disparities in smoking cessation identified in this study [34,35]. CBPR emphasizes the active involvement of community members throughout the research process, ensuring that the perspectives, needs, and cultural contexts of marginalized groups are central to intervention development [36,37] This approach is particularly effective for tackling smoking cessation disparities among Black, Latino, and low-SES individuals by fostering trust, enhancing relevance, and increasing success rates through the integration of lived experiences and local knowledge into program design [38]. Community-engaged strategies can tailor cessation interventions to the specific challenges faced by these populations, such as providing culturally appropriate messaging, accessible resources, and support systems that resonate with community values and norms [39]. By prioritizing partnerships with community organizations and stakeholders, CBPR facilitates the creation of sustainable, effective solutions that empower communities, address structural barriers, and promote equitable health outcomes in smoking cessation efforts [34,35].

One example of a successful community-engaged approach is the Communities Engaged and Advocating for a Smoke-free Environment (CEASE) initiative, developed by Sheikhattari and colleagues [40–44], which has shown promising results in promoting smoking cessation among adults in Baltimore. CEASE is grounded in CBPR principles and involves community members and stakeholders at every stage, from design to implementation and evaluation. This approach recognizes that effective tobacco control must go beyond individual behavior change, addressing broader social and environmental factors such as access to resources, social norms, and economic challenges. In Baltimore, the CEASE approach has been particularly effective due to its focus on culturally tailored interventions that resonate with the target communities [40–44]. The program includes educational workshops, peer support groups, and community advocacy, all designed to empower participants and create a supportive environment for quitting smoking. By leveraging the trust and networks of local organizations, CEASE has successfully reached populations often underserved by traditional cessation programs, such as low-income, Black, and Latino adults [40–44] Continuous feedback loops allow for ongoing refinement of the program based on community input, enhancing its relevance and effectiveness. The success of CEASE in Baltimore demonstrates the potential of community-engaged solutions to reduce smoking-related health disparities and promote healthier communities through sustained, culturally relevant, and community-driven efforts.

The existing literature on smoking cessation extensively documents factors that influence an individual’s likelihood of successfully quitting, including age, nicotine dependence, access to cessation aids, and social support. However, while many studies have explored these factors in general populations, fewer have focused specifically on long-term cessation success among diverse demographic groups [45,46]. Most studies are cross-sectional or have short follow-up periods, limiting their ability to capture the sustained quit success necessary for long-term health benefits. Moreover, while disparities in cessation are acknowledged, a more nuanced understanding of how these disparities manifest and persist over time—particularly in the context of structural barriers that disproportionately affect certain groups—is needed.

The Population Assessment of Tobacco and Health (PATH) study [30,47,48] provides a unique opportunity to address these gaps due to its longitudinal design and nationally representative sample of U.S. adults. By following individuals over an extended period, the PATH study allows for the examination of long-term smoking cessation outcomes across diverse subpopulations. The data collected includes detailed information on smoking behaviors, cessation attempts, and various demographic factors, enabling exploration of the intersection of these variables in ways that previous studies have not. This study leverages PATH data to investigate which demographic groups among daily cigarette smokers are less likely to achieve long-term smoking cessation, with a particular focus on understanding the roles of race, ethnicity, and SES.

Understanding the dynamics of smoking cessation in these groups is essential, as it provides insights into the underlying reasons for observed disparities and highlights potential areas for intervention. For example, males may be less likely to engage in help-seeking behaviors or may perceive smoking as a socially normative behavior that is harder to change. Black and Latino smokers may encounter unique cultural and systemic barriers, such as mistrust of healthcare providers or limited access to culturally tailored cessation resources. Meanwhile, individuals living in poverty and those with lower educational attainment may struggle with health literacy issues, making it challenging to navigate available cessation resources effectively. By examining these groups, the study seeks to identify actionable factors that can be addressed through targeted public health interventions and policies.

### 5.1. Limitations

Several limitations are worth mentioning. First, our measures did not specify the number of cigarettes smoked per day. The study included participants who smoked daily as well as those who smoked on some days per week; however, the exact frequency (e.g., 2, 3, 4, or 5 days per week) was not detailed. Without information on the daily cigarette count and a clear definition of “some days,” making precise comparisons is challenging. Additionally, we did not analyze sex and gender differences in the observed associations. Furthermore, the effects of socioeconomic disparities on smoking cessation may vary across different races, ethnicities, genders, and age groups, among other factors. This study, however, only assessed overall effects without stratifying by these subgroups. Despite these limitations, the study benefits from using data from a nationally representative sample of U.S. adults, ensuring diversity in terms of race, ethnicity, and socioeconomic status (SES). Additionally, the long-term follow-up of participants is a significant strength of this study.

### 5.2. Implications

Tailored public health interventions are necessary to meet the specific needs of groups with lower cessation success [49,50]. Efforts should focus on reducing structural barriers, enhancing access to cessation aids, and providing culturally sensitive support to improve quit rates among male, Black, Latino, and low-educated smokers in the United States. The findings of this study have important implications for public health strategies and policies aimed at reducing smoking-related health disparities in the U.S. By identifying marginalized groups less likely to quit smoking and understanding the factors contributing to these disparities, the study can inform investments in developing tailored and effective cessation interventions in marginalized communities. Interventions should be designed to address the unique challenges faced by racial, ethnic, and low SES minority smokers, such as culturally appropriate cessation programs or enhanced access to cessation aids in underserved communities. Moreover, the study contributes to the broader literature by providing a comprehensive understanding of the long-term dynamics of smoking cessation in a nationally representative sample, thereby filling critical gaps and advancing the field of tobacco control research.

## 6. Conclusion

In summary, this study seeks to deepen our understanding of smoking cessation disparities among daily smokers in the U.S., with the ultimate goal of informing more equitable public health practices and policies. By leveraging robust data from the PATH study, this research sheds light on the complex interplay of demographic factors that influence quit success, offering insights crucial for achieving the broader public health objective of reducing smoking prevalence across all population groups.

## Figures and Tables

**Table 1.** Descriptive Data of Current Smokers that Were Followed for eight Years

	Proportion	SE	95%	CI
Census Region				
Northeast	16.54	0.01	14.34	19.00
Midwest	24.00	0.01	21.60	26.58
South	40.50	0.01	37.91	43.14
West	18.96	0.01	16.99	21.11
Age				
18 to 24 years old	15.48	0.00	14.73	16.27
25 to 34 years old	24.08	0.00	23.19	25.01
35 to 44 years old	18.95	0.00	18.11	19.82
45 to 54 years old	19.46	0.00	18.68	20.27
55 to 64 years old	15.06	0.00	14.27	15.89
65 to 74 years old	5.51	0.00	5.03	6.03
75 years old or older	1.45	0.00	1.21	1.75
Sex				
Female	44.21	0.00	43.29	45.13
Male	55.79	0.00	54.87	56.71
Race				
White	75.11	0.01	72.20	77.81
Black	16.34	0.01	13.95	19.05
Other	8.55	0.01	7.57	9.64
Latino				
No	86.71	0.01	84.89	88.34
Yes	13.29	0.01	11.66	15.11
Education				
Less than high school	16.82	0.00	15.95	17.73
GED	10.68	0.00	10.01	11.39
High school graduate	28.55	0.01	27.45	29.68
Some college (no degree) or associate degree	32.77	0.01	31.71	33.85
Bachelor’s degree	8.57	0.00	7.80	9.41
Advanced degree	2.61	0.00	2.27	2.98
Poverty				
No	60.41	0.01	58.93	61.86
Yes	39.59	0.01	38.14	41.07
Health Insurance				
Some private insurance	49.06	0.01	47.72	50.40
Private insurance, some Medicare	8.92	0.00	8.25	9.64
Private insurance or Medicare, s	13.00	0.00	12.10	13.95
Other insurance only	3.44	0.00	3.09	3.84
No insurance	25.58	0.01	24.58	26.61
				
Successful Quit (wave 1 to wave 6)				
No	91.17	0.00	90.53	91.77
Yes	8.83	0.00	8.23	9.47

**Table 2.** Predictors of successful quit in current smokers at baseline (over 8 years of follow up)

	Odds Ratio	Linearized SE	95%	CI	p
Census Region					
Northeast	Ref				
Midwest	1.15	0.13	0.91	1.44	0.238
South	1.14	0.13	0.90	1.43	0.272
West	1.27	0.16	0.98	1.63	0.067
Age					
18 to 24 years old					
25 to 34 years old	0.72	0.06	0.61	0.86	< 0.001
35 to 44 years old	0.55	0.06	0.45	0.68	< 0.001
45 to 54 years old	0.43	0.05	0.34	0.54	< 0.001
55 to 64 years old	0.46	0.06	0.35	0.59	< 0.001
65 to 74 years old	0.40	0.09	0.26	0.62	< 0.001
75 years old or older	0.22	0.17	0.05	0.97	0.046
Sex (Male)	0.81	0.07	0.68	0.96	0.018
Race					
White	Ref				
Black	1.23	0.11	1.03	1.47	0.020
Other	1.00	0.14	0.76	1.31	0.987
Latino	1.66	0.17	1.35	2.03	< 0.001
Education					
Less than high school	Ref				
GED	0.94	0.13	0.72	1.24	0.669
High school graduate	1.12	0.13	0.89	1.39	0.330
Some college (no degree) or associate	1.22	0.13	0.98	1.51	0.081
Bachelor’s degree or	1.82	0.27	1.35	2.44	0.000
Advanced degree	1.71	0.38	1.10	2.66	0.018
Advanced degree					
Poverty	0.84	0.06	0.73	0.97	0.020
Health Insurance					
Some private insurance	Ref				
Private insurance, some Medicare	0.87	0.13	0.65	1.18	0.375
Private insurance or Medicare	0.84	0.09	0.67	1.04	0.109
Other insurance only	1.01	0.20	0.68	1.51	0.966
No insurance	0.88	0.07	0.75	1.04	0.124
Intercept	0.14	0.02	0.10	0.19	< 0.001
